# Mid-term results after proximal humeral fractures following angular stable plate fixation in elderly patients—which scores can be evaluated by a telephone-based assessment?

**DOI:** 10.1186/s13018-019-1536-8

**Published:** 2020-01-06

**Authors:** Patrick Ziegler, Kim Stierand, Christian Bahrs, Marc-Daniel Ahrend

**Affiliations:** 1 BG- Center for Trauma & Reconstructive Surgery, Eberhard Karls University of Tübingen, 72076 Tübingen, Germany; 2 AO Research Institute Davos, 7270 Davos, Switzerland

**Keywords:** Angular stable plate fixation, Proximal humeral fracture, Fractures in the elderly, Outcome

## Abstract

**Background:**

The aim was to evaluate postsurgical outcome in elderly patients (> 70 years) after open reduction and internal fixation (ORIF) of proximal humeral fractures and compare the test-retest agreement of scores which are frequently used to assess the outcome of upper extremity disorders.

**Methods:**

Ninety patients (78.1 ± 5.2 years) with a minimum follow-up of 2 years (3.7 ± 0.9 years) following angular stable plate fixation of a proximal humeral fracture (2-part: 34, 3-part: 41, 4-part: 12) were enrolled. Two telephone-based interviews assessed Disabilities of the Arm, Shoulder and Hand Score (DASH), Oxford Shoulder Score (OSS), and Constant Score adjusted for interview assessment (CS) by two independent interviewers. Correlations, Bland-Altman analyses, Cross tabulation, and weighted Kappa measure of agreement (*k*) were calculated to assess differences and the test-retest agreement between the categories of each score.

**Results:**

In the first and second interview, we could state fair outcomes: CS 91 (range 40–100) and 65.5 (23–86), DASH 12.5 (0–64.2) and 18.3 (0–66.7), and OSS 58 (33–60) and 55 (25–60) points.

The test-retest correlations were *r* = 0.67, *r* = 0.77, and *r* = 0.71 for CS, DASH, and OSS. Bland-Altman analyses showed absolute mean individual score differences of − 22.3, 4.9, and − 3.0 for CS, DASH, and OSS. Limits of agreement represented possible differences of 21.6%, 15.5%, and 9.0% of CS, DASH, and OSS. The category agreements were medium to high: CS 55.9% (*k* = 0.08), DASH 87.2% (*k* = 0.62), and OSS 99.3% (*k* = 0.74).

**Conclusion:**

Patients showed good subjective outcomes. The test-retest agreement of the interview-adjusted CS was low, but telephone-based assessment of OSS and DASH present as an alternative to collect outcomes in elderly patients.

**Trial registration:**

(250/2011BO2).

## Background

Proximal humeral fractures are common injuries of the elderly patient with an increasing incidence [1–4]. Depending on factors such as patient age, pre-existing conditions, degree of dislocation, fracture morphology, and patient’s expectation, the decision between conservative treatment and surgical intervention is made. However, no consensus about the gold-standard treatment of proximal humeral fractures is present [5–7]. Especially in patients with a multifragmentary fracture and/or severe fracture dislocation, open reduction and internal fixation is often the treatment of choice [8–10]. Overall good results in 70 to 80% of the cases can be achieved with angular stable plates. However, complications such as screw perforation, humeral head necrosis, and secondary fracture displacement are common [11, 12]. So far, long-term results following angular stable plate fixation for proximal humeral fractures are limited in the literature, especially for elderly patients [13–15]..

Patient-reported outcome measures (PROM) are important evaluation tools to assess clinical and functional outcomes from the patient’s perspective [16, 17]. The measurement of surgical outcome parameters and the detection of complications is difficult in elderly trauma patients, because follow-up appointments often represent a barrier for this patient group with immobility and dependency of assisted transportation. Therefore, telephone-based assessments of PROMs can be a time-saving alternative in this patient group. However, no data is available regarding the usefulness as well as the test-retest agreement of telephone-based assessment of standardized scores in proximal humeral fractures of elderly patients.

This cohort study had the primary aim to assess mid-term results using standardized and established scores (DASH Score—Disabilities of the Arm, Shoulder and Hand), OSS (Oxford Shoulder Score), and the interview-based Constant score according to Boehm at al [18]. in patients aged over 70 years with proximal humeral fractures treated with angular stable plate fixation. The secondary aim was to compare the test-retest agreement of the telephone-based assessment of the scores. We hypothesized (1) that the majority of patients had good results at least 2 years after surgery in all three scores and (2) that the DASH and the OSS had higher test-re-test agreement compared to the Constant score regarding the subjective shoulder function in the elderly population.

## Materials and methods

### Study design and patient recruitment

**Fig. 1:**
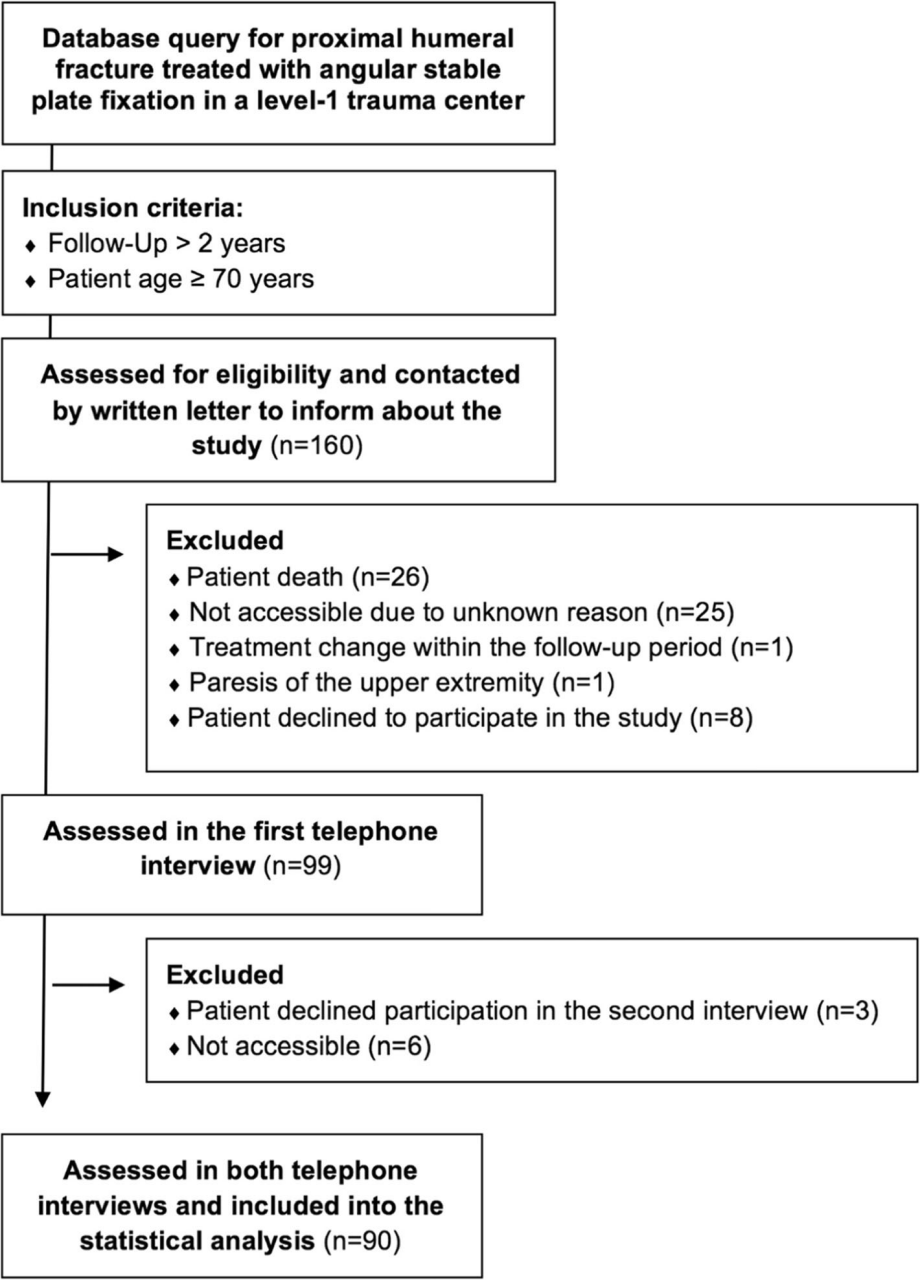
Patient flow chart

The present cohort study (level IV) analyzed functional surgical outcomes of proximal humeral fractures with angular stable plate fixation at least 2 years postoperatively and was approved by the local ethics committee. Inclusion criteria were a minimum age of 70 years and a proximal humeral fracture following ORIF. Exclusion criteria were change of therapeutic concept of an anatomical reconstruction of the humeral head during the follow-up period (e.g., revision surgery with arthroplasty) and non-shoulder-related severe comorbidities (e.g., dementia). Within a 2-year timeframe, 160 consecutive patients with proximal humeral fractures older than 70 years were treated with angular stable plate fixation. All patients were contacted at least 2 years (3.7 ± 0.9 (range 2.3 to 5.4 years) following surgical intervention by written letter for study participations 2 weeks ahead of telephone-based interviewing. A second interview-based score assessment was performed 3 months after the first interview within a 2-week period. The first interview was conducted by an experienced physician assistant. The second interview was conducted by a medical doctor (resident). Both interviewers were blinded about the pre-, peri- and postoperative details and were not involved in the surgeries of the patients. Both interviewers were experienced in data collection for research projects more than 3 years. Both interviewers were educated by the same senior consultant to have higher interview standardization. The patient flow chart is presented in Fig. 1. The work has been reported in line with the STROCSS criteria [19].

### Participants

The final data set comprised 90 patients (male: 12, female: 78; height: 164.8 ± 7.2 cm; weight: 68.9 ± 17.8 kg) with a mean age of 78.1 ± 5.2 years (range 70.1 to 89.8 years). According to the Neer classification [20], the cohort consisted of 34 2-part fractures, 41 3-part fractures, and 12 4-part fractures. According to the AO/OTA classification [21], these fractures were classified as 34 A, 32 B, and 21 C fractures. No traumatic nerve injury or vascular comorbidities occurred. Fractures were treated in 28.9% of the cases with a PHILOS® plate (DePuy Synthes, West Chester, Pennsylvania, USA) and in 71.1% of the cases with a WINSTA-PH WS proximal Humerus (Axomed GmbH, Freiburg, Germany).

### Surgical treatment and postoperative care

Patients were treated using either the PHILOS® plate or the WINSTA-PH WS® proximal humeral plate. The operations were performed by using either a deltoid split, a deltoideo-pectoral, or an anterolateral approach according to Bigliani. Extensive fracture exposure was avoided trying to perform the surgery as less invasive as possible [22]. The fracture was reduced by indirect maneuvers, with the help of k-wires and/or an elevatorium or with bone hooks to reduce the tuberosities. As described by Bahrs et al. [13], the plate was placed at least 5 to 8 mm distal to the upper end of the greater tuberosity and 2 to 4 mm lateral to the bicipital groove. Positioning of the plate and screws were controlled intraoperatively by an image intensifier.

Following surgery, the shoulder was immobilized using a shoulder sling during the first 7 days. Afterwards for 4 weeks, active-assisted movement including up to 90° of abduction and flexion was allowed. All patients received physiotherapeutic treatment after the operation.

### Outcome variables

The interview-based Constant score according to Boehm et al. [18], the DASH score, and the Oxford Shoulder Score were assessed during both telephone-based interviews. The Constant score assesses pain and shoulder function during daily activities, range of motion, and shoulder strength [23]. The adjusted Constant Score according to Boehm et al. [18] evaluates the strength of the shoulder with the help of housewares (e.g., water bottles, sugar packages) with a defined weight. These weights had to be held in front of the body without leaning against a wall in 90° flexion in the shoulder and with an extended arm for at least 5 s. The Constant score was categorized as excellent from 100 to 86 points, good from 71 to 85 points, satisfying from 70 to 56 points, and worse lower than 56 points [24]. The DASH score is a subject patient outcome score with 30 items regarding symptoms and functionality of the upper limb. The final score is calculated based on a standardized formula and ranges from 0 (no disability) to 100 (most severe disability) [25]. Score categories were excellent and good from 0 to 15 points, satisfying from 16 to 40 points, and a worse result with more than 40 points [26]. The OSS includes questions about pain, sleep, and daily life activities and ranges from 12 to 60 points. Outcome measured with the OSS is categorized in poor from 12 to 20 points, satisfying from 21 to 30 points, good from 31 to 40, and very good between 41 and 60 points [27].

### Statistical analysis

The continuous scores were described descriptively as mean (median; range; minimum-maximum). Categories of scores, implant-related complications, as well as radiographic parameters were described as *n* (%). Shapiro-Wilk test was used to check if data was normally distributed. As scores were not normally distributed, correlations between the first and the second interview were computed with Spearman’s rank correlation coefficient. The coefficients were analyzed using a scale proposed by Hopkins [28]: correlation coefficient < 0.1, trivial relationship; 0.1–0.3, low; 0.3–0.5, moderate; 0.5–0.7, strong; 0.7–0.9, very strong; > 0.9, nearly perfect. Cross tabulation was applied and weighted Kappa measure of agreement (*k*) was calculated to assess the test-retest agreement of the categories of each score. Weighting was done in accordance to the above described categories for each score: trichotomized (0, 0.5, 1) DASH score, as well as quatrochotomized (0, 0.33, 0.67, 1) OSS and Constant score. Bland and Altman-analyses [29] were calculated to describe mean differences and limits of agreement between the two interviews for each score. A two-sided *p*-value of < 0.05 was considered statistically significant.

## Results

Score values of the first and the second interview assessed by the physician assistant and the medical doctor are summarized in Table 1. The OSS differed with a mean score of 55.7 points (median 58; range 33–60) in the first interview and 52.7 points (median 55; range 25–60 points) in the second interview. The mean DASH score was 14.7 points (median 12.5; range 0–64.2) in the first interview and 19.6 points (median 18.3; range 0–66.7) in the second interview. The mean adjusted Constant score according to Boehm et al. [18] was 86.5 (median 91; range 40–100) points in the first interview and 64.2 (median 65.5; range 23–86) points in the second interview.

**Table 1:** Summary of score assessments (mean (median; range)). A negative value of individual differences between interviews means that the score was lower in the second interview

	First score assessment	Second score assessment	Individual differences between interviews	Spearman’s correlation	Weighted *k* measure of agreement (categories)	Agreement between interviews (categories)
OSS	55.7 (median: 58; 33–60)	52.7 (median: 55; 25–60)	− 3.0 (median: − 2; − 17–5)	0.71	0.74	99.3%
DASH	14.7 (median: 12.5; 0–64.2)	19.6 (median: 18.3; 0–66.7)	4.9 (median: 2.5; − 14.2–31.7)	0.77	0.62	87.2%
Constant	86.5 (median: 91; 40–100)	64.2 (median: 65.5; 23–86)	− 22.3 (median: − 21; − 53–10)	0.67	0.08	55.9%

*OSS* Oxford Shoulder Score; *DASH* Disabilities of the Arm, Shoulder and Hand-Score; *Constant* Constant score

**Fig. 2:**
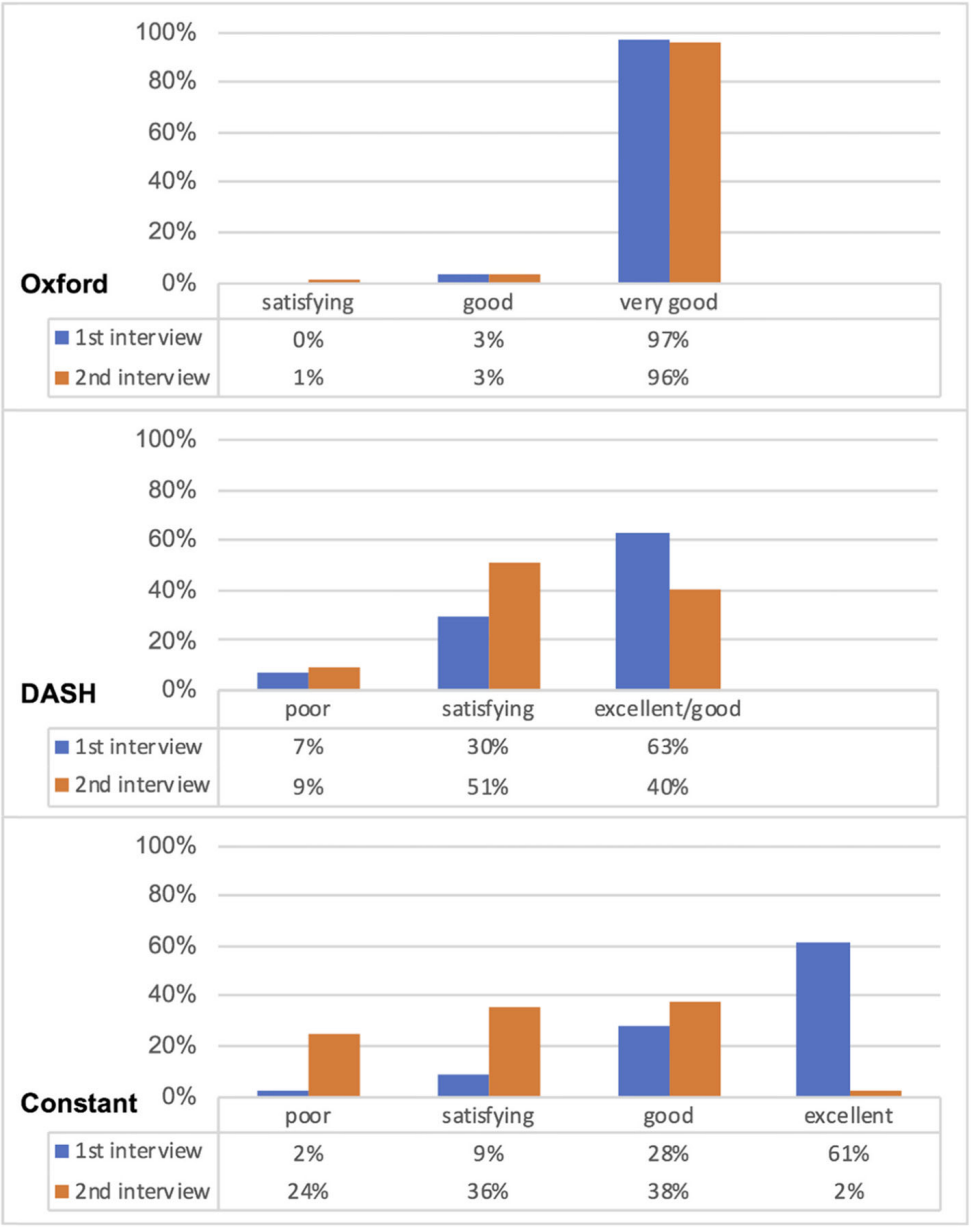
Changes of the categorized results of the Oxford, DASH, and Constant score between the first (blue) and second (orange) interview

The categories of the individual scores are displayed in Fig. 2. The figure shows that the majority of patients reached in the first interview of the Constant score an excellent result (*n* = 55; 61%) and in the second interview a good result (*n* = 34; 38%). In the OSS, nearly all patients had a very good result in the first (*n* = 87; 97%) and second (*n* = 86; 96%) interview. The category of the DASH score was in the majority of patients ‘excellent/good’ in the first interview and ‘satisfying’ in the second interview.

### Test-retest agreement

**Fig. 3:**
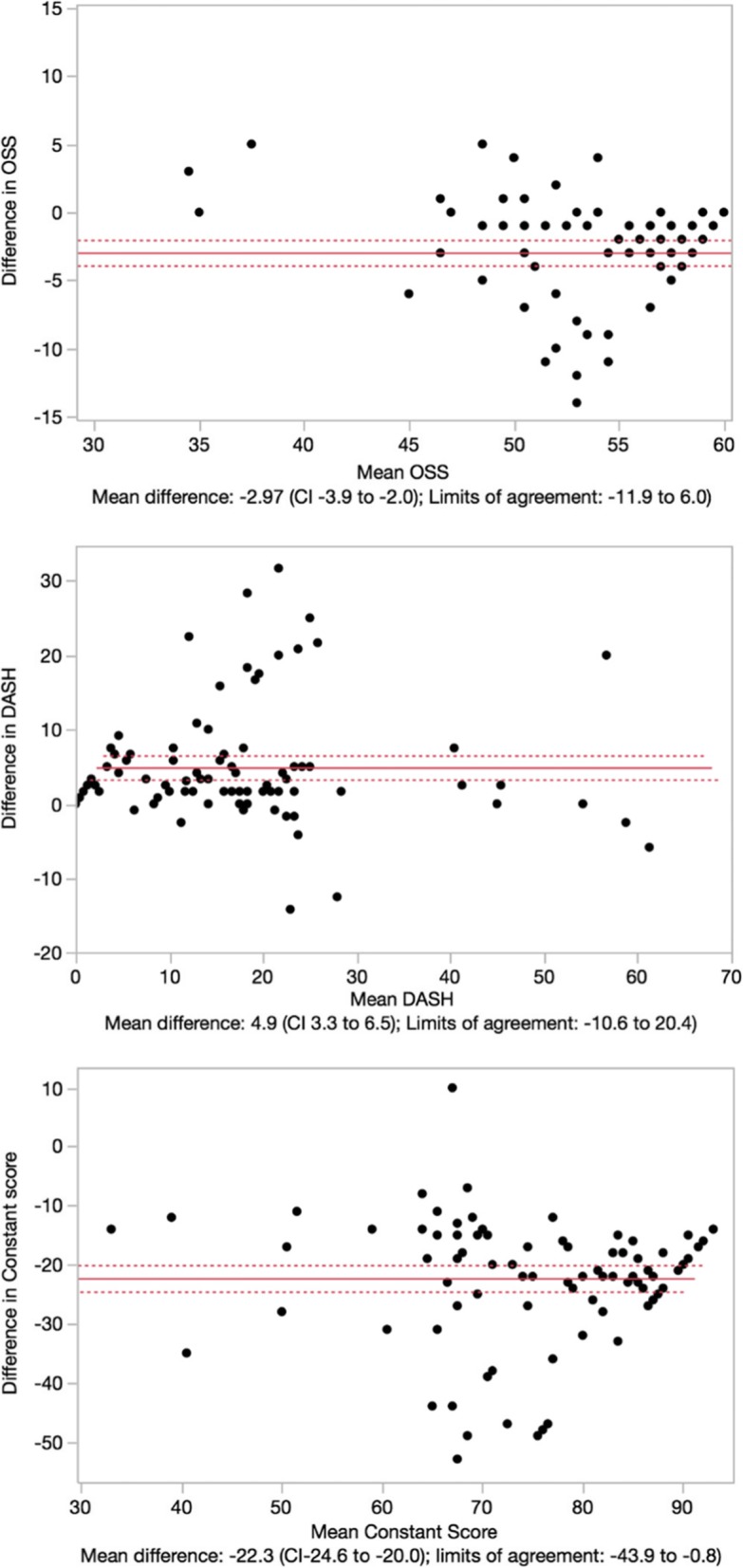
Bland-Altman analyses showing plots with 95% CI and 95% limits of agreement of the Oxford shoulder score (OSS), the DASH score, and the Constant score

The correlations between test and retest were strong to very strong. The correlation coefficients (Spearman) were *r* = 0.67 (*p* < 0.0001) for the Constant score, *r* = 0.77 (*p* < 0.0001) for the DASH, and *r* = 0.71 (*p* < 0.0001) for the OSS. The agreement of categories between the first and the second interview were 55.9% (weighted *k* = 0.08) for the Constant score, 87.2% (weighted *k* = 0.62) for the DASH score, and 99.3% (weighted *k* = 0.74) for the OSS (all *p* < 0.001). Bland-Altman analyses (see Fig. 3) showed significant systematic changes in scores indicated by 95%CI which did not include 0. Moreover, limits of agreement of all three scores, especially for the Constant score, showed large ranges indicating low individual score agreement.

### Radiographic evaluation

Anatomic reduction postoperatively was achieved in 55 (63.2%) cases. Twenty-four (27.6%) patients had either a displacement of the tuberosity, a varus/valgus angulation on the AP view of ± 15°, or an anteversion/retroversion on the axillary view of ± 15°. Eight (9.2%) patients had a displacement of the tuberosity and/or a varus/valgus angulation and an anteversion/retroversion postoperatively.

## Discussion

The primary aim of the present study was to describe patient-reported and clinical outcomes following angular stable plate fixation of proximal humeral fractures in patients over 70 years of age. As hypothesized, most patients showed good to excellent subjective outcomes at least two years following surgical treatment. Moreover, retrospective analysis of postoperative radiographs documented satisfying results with sufficient reduction of the fracture in most of the patients.

Most published studies analyzed short-term outcomes or reported about cohorts with patients younger than 70 years old. In the meta-analysis by Dai et al. [30], included studies reported Constant scores between 65.2 and 83.9 points between 6 and 60 months following locking compression plate fixation of proximal humeral fractures [31, 32]. Ockert et al. [14] reported about 43 fractures with an average age of 67.6 year and a median follow-up of 10 years. Patients reached 75.3 points in mean in the Constant score and the majority showed good to excellent outcomes. However, 16% of the patients had a poor long-term outcome [14]. Fifty-seven patients aged 65 ± 14 years were analyzed by Hirschmann et al. [33]. Four to 6 years postoperatively, patients reached 15.3 ± 17.7 points in the DASH score and 70.5 ± 17.7 points in the Constant score. In a prospective evaluation of 77 patients (mean age, 62 years) with a mean follow-up period of 96 months, Bahrs et al. [13] found a mean Constant and DASH score of 79 points and 12 points. Schulte et al. [34] found in 43 patients (average age, 68 years) with proximal humeral fractures treated with a locking compression plate a mean DASH score of 11 points (range 0–21.7). Plath et al. [35] compared patients with a proximal humeral fracture older than 60 years who were treated with a locking blade nail or a locking plate (PHILOS). One year postoperatively, patients treated with a locking plate reached a median Constant score of 64 ± 20 points and a DASH score of 42 ± 19 points.

A poor functional outcome as well as higher risk of screw-related complications following plate fixation is highly related to age and gender [13, 14] [36, 37]. Our sample consisted of patients older than 70 years with a mean age of 78.1 years. In the first and second assessment of our study, the Constant score was 86.5 points and 64.2 points, the DASH score 14.7 and 19.6 points, and 55.7 and 52.7 points in the OSS. Despite the older sample of the present study, our results underlined the benefits of surgical intervention. Scores showed similar values as studies with younger patients [33, 38].

The secondary aim of our study was to evaluate the assessment of telephone-based interviewing in a blinded test-retest analysis. As hypothesized, the agreement of the telephone-based assessments of the functional impairment reported in the interview-adjusted Constant score is low. The agreement between categories (55.9%) was limited as well as high mean individual absolute differences between the interviews. These differences of the Constant score were mostly related to different answers about pain free mobilization as well as measurement of muscle strength. Both categories account for 65% of the total score. Subjective parameter such as pain and daily life activities account for smaller proportions. The measurement of muscle strength and mobilization was adapted by the interview-adjusted Constant score according to Boehm et al. [18]. It was reported as a reliable and valid tool to assess the clinical outcome in patients who cannot attend a follow-up appointment. However, this requires high compliance of patients and accurate instructions by the interviewer.

We could only find small differences between the first and the second assessment of the DASH and the OSS. Despite higher test-retest agreements, both scores do not analyze the objective functionality and mobility of the shoulder as the Constant score. Additionally, the Bland-Altman method revealed that also the DASH and the OSS were limited in the absolute individual score agreement. Limits of agreement represent possible differences of approximately 15.5% and 9.0% of their respective scoring scales. However, high agreement between the categories of the DASH (87.2%) and the OSS (99.3%) were found for these scores.

Previously published by Mahabier et al. [16], the validity and reliability of outcome evaluation over time was reported for the DASH and the Constant score in humeral shaft fractures. The sample consisted of 140 patients with a median age of 58 years during a 1-year postoperative interval. In accordance to our results, the reliability of the DASH score was higher than for the Constant Score [16]. Slobogean et al. [39] found smaller mean differences in the Bland-Altman analysis for the DASH score (mean difference, 0.4 (95% CI − 2.3 to 3.1)) compared to our study. However, similar limits of agreement (− 15.2 to 15.9) were found. In our study, interviews were conducted from two different observers: a physical assistant and a medical doctor. Differences between the scores could have also been influenced by inter-observer differences of the education, personality, motivation, and how detailed instructions were made [40]. However, using two observers guaranteed blinded data collection. Results are limited as no inter-observer reliability was assessed.

Further limitations of the present study relied on the study design. Telephone-based interviews are a useful alternative to evaluate and monitor surgical outcomes, especially the DASH and the OSS, and to detect possible complications with low costs, easier and faster assessment of the interview, and lower lost-to-follow-up rates. Our follow-up rate (56.3%) was higher compared to the follow-up rates of previous studies, e.g., Bahrs et al. [13] (40%) or Ockert et al. [14] (35%). Patient’s death was the main reason of lost-to-follow-up. Despite lower lost-to-follow-up rates, the assessment of functional outcomes is difficult via phone calls, especially in the elderly population and answers of patients could not be verified. Following our study results, we do not recommend using the CS in a telephone-based assessment of shoulder function in elderly patients. An additional appointment in our clinic could have addressed this issue and would have decreased the re-call bias. Interview skills and differences in interview assessment can highly influence interview results. We used standardized scores which were validated in several studies. To reduce differences occurred by the different interviewer, we paid attention to strictly stick to a standardized interview protocol (order of questions etc.). Both interviewers were educated by the same senior consultant who supervised five telephone-based interviews, each.

In our study, the interviews were conducted from two different observers at two different time points: a physical assistant and a medical doctor. Differences between the scores could have also been influenced by inter-observer differences of the education, personality, motivation, and how detailed instructions were made. However, using two observers guaranteed blinded data collection. Results are limited as no inter-observer reliability was assessed.

## Conclusion

Most patients reported about good subjective outcomes at least 2 years following surgical treatment. The test-re-test agreement of the clinical outcomes measured with an interview-based CS was low. Only small differences and high agreement between score categories were found for DASH and OSS. Despite higher test-retest agreement, both scores do not analyze the functionality and mobility of the shoulder as detailed as the Constant score. Telephone-based assessment of OSS and DASH presents as an alternative to collect and monitor surgical outcomes in elderly patients with low costs, easier assessment, and lower lost-to-follow-up rates.

## Data Availability

The datasets used and analyzed during the current study are available from the corresponding author on reasonable request.

## Abbreviations

AO: Arbeitsgemeinschaft für Osteosynthesefragen
DASH: Disabilities of the Arm, Shoulder and Hand
OSS: Oxford Shoulder Score
